# Associations between test anxiety and academic achievement in distance education: avoidance- and overcontrol-related process and the moderating role of exam format preference

**DOI:** 10.3389/fpsyg.2026.1865966

**Published:** 2026-09-04

**Authors:** Münevver Başman

**Affiliations:** Department of Measurement and Evaluation in Education, Marmara University, Maltepe, Türkiye

**Keywords:** academic perfectionism, academic procrastination, distance education, exam format preference, test anxiety

## Abstract

**Introduction:**

The increased use of distance education has heightened the importance of understanding the self-regulatory correlates of student academic success. Although test anxiety has generally been associated with lower academic achievement, the behavioral and dispositional variables statistically linking these constructs remain insufficiently understood, particularly in digitally mediated learning environments. Grounded in Situated Expectancy-Value Theory and Self-Regulated Learning theory, this study examined the direct and indirect associations between test anxiety and cumulative GPA through academic procrastination and academic perfectionism and investigated whether these indirect associations differed according to students’ self-reported exam format preference.

**Methods:**

A cross-sectional correlational design was employed with 928 students enrolled in distance education programs. Structural equation modeling was first used to assess the measurement model and estimate the hypothesized mediation model. Subsequently, Conditional Process Analysis (PROCESS Model 58) was employed to test the hypothesized moderated mediation model.

**Results:**

Academic procrastination and academic perfectionism partially mediated the association between test anxiety and cumulative GPA. Furthermore, exam format preference significantly moderated the indirect association through academic procrastination, although the effect was small. Specifically, the negative association between academic procrastination and GPA was weaker among students who preferred constructed-response formats than among those who preferred multiple-choice formats. In contrast, the indirect association through academic perfectionism did not vary by exam format preference.

**Discussion:**

These findings extend the literature by integrating emotional, self-regulatory, and individual-difference variables within a unified framework. They highlight the roles of academic procrastination and academic perfectionism in the association between test anxiety and academic achievement and suggest that exam format preference may modestly shape the procrastination-related pathway. Overall, the findings provide a more comprehensive understanding of academic achievement in distance education.

## 1 Introduction

Many students in distance education have less structured support systems for regulating their thoughts, behaviors, and emotions. This results in the learner having a larger share of the responsibility for managing both their behavior related to the course and their test-taking anxiety. Therefore, apart from offering flexibility and educational access to numerous students, distance education also complicates their capacity to regulate their psychological processes, highlighting the growing importance of understanding how students regulate perceived costs and effort. Despite its rapid expansion, student achievement in these settings may be shaped by a range of contextual and psychological factors that are not yet fully understood, and prior evidence suggests that outcomes in online and distance formats can differ from those in face-to-face settings (Gopal et al., 2021; Xu and Jaggars, 2013). Consequently, there is an increasing focus on examining the relationships among psychological factors, learning contexts, and academic achievements, particularly within environments marked by a scarcity of external structure and assistance. Self-regulated learning theory suggests that in low-scaffolding environments, students’ capacity to monitor and direct their own learning behaviors may become particularly important for academic outcomes (Zimmerman, 2000). Understanding how emotional states such as test anxiety disrupt self-regulatory processes is therefore particularly relevant to distance education research.

Although there is a significant amount of evidence supporting a negative correlation between test anxiety and academic achievement, most of these studies have primarily focused on the direct association between test anxiety and academic achievement (Cassady and Johnson, 2002; Eysenck et al., 2007; Thomas et al., 2017; von der Embse et al., 2018) and do not provide an adequate explanation of how anxiety may be related to a student’s ability to complete assignments or take exams. Recent research suggests that this relationship may be better understood by considering self-regulatory processes, including academic procrastination and academic perfectionism. Procrastination is frequently conceptualized as a coping strategy associated with the regulation of immediate anxiety, whereas perfectionism has been associated with heightened concern over mistakes and rigid, self-imposed performance standards (Flett and Hewitt, 2002; Frost et al., 1990).

Despite extensive research on self-regulatory processes, prior models have largely treated procrastination and perfectionism in isolation, overlooking how avoidance- and overcontrol-related self-regulatory processes may jointly characterize the association between test anxiety and academic achievement. Furthermore, while the association between test anxiety, self-regulatory processes, and academic achievement may vary across learning contexts, few studies have explored students’ orientations toward different exam formats as a potential moderator of these associations. Students may differ in their preferences for examination formats, and these preferences are likely to reflect multiple influences rather than a single motivational construct. Previous research has shown that different examination formats vary in their cognitive and strategic demands (Bridgeman, 1992; Rodriguez, 2003). Nevertheless, prior models have rarely examined self-reported exam format preference as an individual-difference moderator of the associations between anxiety-related self-regulatory processes and academic achievement.

In order to address some of the limitations described above, the current study proposes a theoretically based, context-sensitive model of academic achievement that includes a moderated mediation model. Specifically, the association between test anxiety and academic achievement (GPA) is hypothesized to be mediated by both academic procrastination (avoidance) (Kim and Seo, 2015; Steel, 2007) and academic perfectionism (over-control) (Flett and Hewitt, 2002) and moderated by exam format preference. In this study, academic perfectionism refers specifically to maladaptive aspects of perfectionism, such as chronic self-doubt, maladaptive social comparison, and the pursuit of unrealistically high or rigid standards (Chung and Shin, 2024; Flett and Hewitt, 2002; Frost et al., 1990; Odacı et al., 2017). Within the proposed theoretical framework, these academic perfectionistic concerns are considered theoretically relevant as a domain-specific self-regulatory process that may become particularly salient in the context of heightened test anxiety.

By examining this theoretically specified model, the current study aims to move beyond a static, direct-association approach to understanding academic achievement by considering emotional costs, self-regulatory processes, and contextual conditions within an integrated framework. Overall, this approach expands the field of study of test anxiety and academic achievement by focusing not only on whether test anxiety is associated with academic achievement, but also on how this association is reflected in multiple self-regulatory processes and whether these associations vary according to students’ exam format preferences.

This study reconceptualizes the association between test anxiety and academic achievement by considering two qualitatively distinct self-regulatory processes: avoidance-based regulation, represented by academic procrastination, and overcontrol-based regulation, represented by academic perfectionism. This dual-process framework suggests that the association between test anxiety and academic achievement may be better understood by considering distinct forms of self-regulatory difficulty rather than a single self-regulatory process. Building on this perspective, the study makes three contributions. First, it integrates avoidance-based and overcontrol-based self-regulatory processes as complementary processes within a unified Situated Expectancy-Value Theory (SEVT) and Self-Regulated Learning theory (SRL) framework, moving beyond models that treat procrastination and perfectionism in isolation. Second, it introduces self-reported exam format preference as an individual-difference moderator, examining whether the associations between anxiety-related self-regulatory processes and academic achievement differ between students who prefer multiple-choice and constructed-response examinations. Third, it applies this integrative model to a distance education context, where reduced external scaffolding may increase reliance on self-regulatory processes. The study does not assume that these associations are unique to distance education but rather examines them within this educational setting.

The proposed model is developed using Situated Expectancy-Value Theory (SEVT; Eccles et al., 1983; Eccles and Wigfield, 2002, 2020), which posits that students’ achievement-related behaviors are shaped by their expectancy beliefs and perceived costs. Within this framework, test anxiety is viewed as a perceived cost of engaging in evaluative activities. Academic procrastination reflects a cost-avoidance regulatory strategy whereas academic perfectionism represents a cost-amplification and overcontrol response to perceived failure. The present study employs SEVT as an interpretive lens rather than subjecting all three of its components to direct empirical test, with particular focus on the perceived cost construct and its behavioral consequences. For instance, perfectionistic students, driven by fear of failure or excessively high standards, may spend disproportionate time on tasks or avoid them altogether, leading to incomplete assignments or missed deadlines, which are common challenges in online learning contexts (Broadbent and Poon, 2015; Fabian et al., 2022).

Additionally, students’ preferences for different examination formats may be relevant to how they experience evaluative situations. However, the present study does not treat exam format preference as a direct measure of expectancy beliefs, perceived controllability, anticipated success, or perceived costs. Instead, consistent with SEVT (Eccles and Wigfield, 2020), the theory is used as a broad interpretive framework for considering why the association between self-regulatory processes and academic achievement might differ between students reporting different examination-format preferences. Accordingly, exam format preference is examined as a self-reported individual-difference moderator rather than as an operational measure of a specific SEVT construct.

## 2 Literature review and theoretical framework

### 2.1 Test anxiety and academic achievement

Test anxiety is a multifaceted conceptualization that includes cognitive worry, emotional distress and physiological arousal experienced in evaluation contexts. The components of test anxiety have been shown to impair academic achievement by disrupting attentional control and overloading working memory, therefore reducing task efficiency during exams (Eysenck et al., 2007). This cognitive-interference perspective suggests that students with higher levels of test anxiety are likely to experience lower academic achievement across various indicators including GPA and standardized test scores (von der Embse et al., 2018).

Although there is robust evidence for the association between test anxiety and academic achievement, existing research has primarily utilized direct effect models that offer limited insight into the processes through which anxiety translates to performance outcomes (von der Embse et al., 2018). Importantly, these cognitive interferences are not confined to the testing moment alone but may extend to students’ preparatory behaviors, and shape how they engage with academic tasks prior to evaluation. This perspective is further supported by longitudinal evidence demonstrating that the relationships among motivational beliefs, test anxiety, and academic performance are better understood across multiple stages surrounding examinations rather than at a single point in time. For example, Roick and Ringeisen (2017) showed that self-efficacy, expectancy beliefs, perceived examination importance, test anxiety, and subsequent academic performance formed a temporally ordered process across repeated measurement occasions before and after an examination. Their findings emphasize that understanding test anxiety requires consideration of when these processes occur, not merely whether they are statistically associated. This limitation may be particularly relevant in distance education contexts, where reduced external structure is generally thought to place greater demands on students’ self-regulation (Broadbent and Poon, 2015). Therefore, understanding the association between test anxiety and academic achievement may benefit from moving beyond static outcome models to models that consider the potential roles of behavioral factors, particularly within self-regulated learning frameworks that emphasize students’ active role in managing their own cognitive and emotional resources (Zimmerman, 2000).

Recent evidence from online learning environments further supports this perspective by demonstrating that anxiety is associated not only with examination performance but also with students’ observable learning behaviors. Using Learning Management System (LMS) interaction data from 2,994 online distance learners, Taşkın and Kokoç (2025) found that test anxiety weakened the positive association between online behavioral engagement and academic achievement. Unlike studies relying exclusively on self-reported engagement, their study showed that anxiety-related disengagement was reflected in objectively recorded learning behaviors. These findings complement the present conceptualization of academic procrastination as an avoidance-based self-regulatory process by suggesting that anxiety-related avoidance may be reflected not only in students’ self-reported procrastination but also in objectively observable patterns of online behavioral engagement.

### 2.2 Academic procrastination in the anxiety and achievement processes

Academic procrastination is one of the key self-regulatory processes that may help explain the association between test anxiety and academic achievement. Research shows a consistent relationship between procrastination and test anxiety, although the direction of this relationship remains unclear and may be bidirectional (Onwuegbuzie, 2004). More recently, researchers have begun to view procrastination as a form of emotion regulation rather than simply a failure to manage time. Procrastination is viewed as a means to avoid immediate psychological discomfort by delaying aversive tasks (Sirois and Pychyl, 2013). From this perspective, academic procrastination can be seen as an avoidance response to evaluative threat. Students often delay academic tasks because those tasks evoke fears of failure, negative self-evaluations, and potential distress. While avoiding the task initially reduces anxiety, it ultimately results in reduced preparedness and lower performance. Meta-analyses and experimental studies consistently demonstrate that procrastination is negatively related to academic achievement and positively related to stress and negative outcomes (Kim and Seo, 2015; Tice and Baumeister, 1997).

Procrastination also serves as a mediator between anxiety and performance (Kim and Seo, 2015; Onwuegbuzie, 2004). By limiting the amount of time spent engaging with learning material, procrastination results in students entering evaluative situations underprepared. From a self-regulated learning perspective, procrastination represents a failure in the forethought phase of self-regulation, where students do not adequately plan or initiate academic tasks (Zimmerman, 2000). Therefore, from the standpoint of an anxiety-achievement model, academic procrastination can be conceptualized as an avoidance-based self-regulatory process associated with test anxiety and academic achievement. This avoidance process may also be reflected in students’ actual online learning behaviors. Consistent with this interpretation, Taşkın and Kokoç (2025) showed that test anxiety was associated with reduced behavioral engagement derived from LMS interaction data, suggesting that anxiety-related avoidance is observable not only through self-report but also through objective indicators of participation in online learning environments.

### 2.3 Academic perfectionism in the anxiety and achievement processes

Academic perfectionism represents another key self-regulatory process that is theoretically relevant to the association between test anxiety and academic achievement, particularly with respect to its maladaptive dimensions. Although perfectionistic concerns have often been conceptualized as relatively stable individual differences, their expression in academic settings may become particularly pronounced under conditions of heightened evaluative anxiety. Accordingly, the present study conceptualizes academic perfectionism as domain-specific perfectionistic concerns that may become especially salient in evaluative contexts characterized by heightened test anxiety, rather than as a stable personality trait. Academic perfectionism refers to a tendency to hold excessively high expectations and to evaluate one’s own performance critically. Contemporary conceptualizations distinguish between perfectionistic strivings, which may be adaptive, and perfectionistic concerns, which have been consistently linked to anxiety, mental health problems, and maladaptive academic outcomes (Stoeber and Otto, 2006). Accordingly, the present study focuses on perfectionistic concerns rather than perfectionistic strivings. Although the instrument is titled the Academic Perfectionism Scale, its dimensions (self-doubt, comparison, and idealization) are conceptually more closely aligned with the self-critical and evaluative characteristics of perfectionistic concerns than with the achievement-oriented features of adaptive perfectionistic strivings. Therefore, in the present study, higher scores on the scale were interpreted as reflecting greater maladaptive perfectionistic tendencies in academic contexts. Consistent with this theoretical perspective, academic perfectionism was conceptualized at the construct level rather than in terms of the unique effects of its individual dimensions. Specifically, the latent construct was intended to represent the shared variance across the dimensions of self-doubt, comparison, and idealization, thereby capturing the common maladaptive features of academic perfectionism that correspond to perfectionistic concerns and are central to the proposed theoretical model. Students with elevated perfectionistic concerns, particularly concern over mistakes and doubts about one’s own actions, have been shown to report higher levels of math anxiety, leave more questions unanswered under evaluative pressure, and exhibit lower academic performance (Núñez-Peña and Bono, 2021).

In terms of regulation, academic perfectionism can be understood as a form of overcontrol in reaction to evaluative threats. When students are sensitive to making mistakes, they are inclined to view small errors as indications of failure, increasing their sense of performance pressure and perpetuating evaluative worries (Flett and Hewitt, 2002). Excessive monitoring of performance may be associated with reduced cognitive flexibility and poorer academic performance, especially under high-stakes conditions. Within a self-regulated learning framework, perfectionism may be conceptualized as reflecting dysregulation during the self-reflection phase, in which maladaptive self-evaluation may undermine rather than support future performance (Zimmerman, 2000). Research has repeatedly demonstrated a link between perfectionistic concerns, anxiety, and lower academic and psychological outcomes (Chung and Shin, 2024; Núñez-Peña and Bono, 2021; Stoeber and Otto, 2006). However, academic perfectionism is not merely associated with test anxiety; rather, it can be conceptualized as a distinct self-regulatory process within the theoretical model linking test anxiety and academic achievement. Unlike procrastination, which reflects disengagement from the task, academic perfectionism reflects excessive self-monitoring and rigid evaluative concerns. While procrastination reduces preparatory engagement, perfectionism may impair performance through overcontrol, rigid expectations, and sustained evaluative concern. Therefore, within the framework of anxiety and achievement, academic perfectionism should be viewed as a complementary overcontrol-based self-regulatory process that is theoretically relevant to the association between test anxiety and academic achievement.

### 2.4 The role of exam format preference in testing contexts

Individual differences in cognitive, emotional, and motivational processes are important predictors of academic outcomes. Beyond the structural characteristics of examinations, students may also differ in their subjective preferences for particular examination formats. In the present study, exam format preference is conceptualized as a self-reported individual-difference variable reflecting students’ preference for either multiple-choice or constructed-response examinations. Rather than representing a specific construct within Situated Expectancy-Value Theory (SEVT; Eccles and Wigfield, 2020), exam format preference is examined as an individual characteristic whose moderating role is interpreted within the broader SEVT framework. Exam format preference may be associated with students’ subjective orientations toward evaluative situations, although it should not be interpreted as a direct measure of expectancy beliefs.

Constructed-response and multiple-choice examinations, the two formats most commonly used in educational testing, differ in their cognitive and strategic demands. Constructed-response examinations primarily require retrieval, elaboration, and the organization of knowledge, whereas multiple-choice examinations rely more heavily on recognition and elimination strategies (Bridgeman, 1992; Rodriguez, 2003). These differences may shape how students perceive the difficulty, controllability, and risk associated with each format, and consequently, how anxiety-related self-regulatory processes are associated with academic achievement.

These format-related demands may not weigh equally on the two self-regulatory processes examined in this study. Avoidance-based processes such as procrastination may be particularly sensitive to exam format preference. One possible explanation is that, perceived difficulty and controllability could plausibly differ across formats in ways that affect whether disengagement carries a meaningful performance cost, and that students who prefer different examination formats may differ in their expectations, prior experiences, or perceived fit with particular forms of assessment (Collignon et al., 2024). However, because all participants in the present study completed multiple-choice examinations, these interpretations remain speculative and should not be interpreted as evidence of the effects of examination format itself. Overcontrol-based processes such as academic perfectionism, by contrast, may be less sensitive to exam format preference: perfectionistic concerns involve self-doubt, heightened sensitivity to errors, and rigid performance standards that may remain salient across different evaluative preferences. On this basis, the moderating role of exam format preference is expected to be more relevant for the association involving academic procrastination than for the association involving academic perfectionism.

Accordingly, exam format preference is modeled as a moderator, while SEVT provides the theoretical framework for interpreting these potential differences rather than treating exam format preference as a direct measure of expectancy beliefs or task value. It is expected to moderate the indirect associations between test anxiety and academic achievement via academic procrastination and via academic perfectionism.

### 2.5 Theoretical framework: situated expectancy-value theory and self-regulated learning

The theoretical foundation of the proposed model draws primarily on Situated Expectancy - Value Theory (SEVT; Eccles et al., 1983; Eccles and Wigfield, 2002; Eccles and Wigfield, 2020), which explains achievement related behaviors through three core constructs: (a) expectancy beliefs, referring to individuals’ assessments of their likelihood of success on a given academic task; (b) subjective task value, reflecting the perceived importance, interest, and utility of the task; and (c) perceived costs, encompassing the emotional burden, time investment, and anticipated consequences of failure associated with task engagement. The present study does not aim to provide a comprehensive test of all three SEVT components; rather, SEVT is employed as an interpretive lens to contextualize the role of perceived costs and their translation into anxiety-driven behavioral regulatory behaviors.

Within this framework, test anxiety is conceptualized as a perceived cost reflecting the anticipated emotional and cognitive burden associated with evaluative situations. Elevated perceived costs, according to SEVT, are likely to disrupt the way students regulate their engagement with academic tasks. Academic procrastination represents a cost-avoidance regulatory response, in which students delay task engagement in order to minimize immediate psychological discomfort (Sirois and Pychyl, 2013). Academic perfectionism, by contrast, represents a cost-amplification and overcontrol regulatory response, in which students become highly sensitized to potential errors and engage in excessive performance monitoring (Flett and Hewitt, 2002; Frost et al., 1990). Together, these two regulatory responses constitute complementary processes that are theoretically relevant to the association between perceived costs, operationalized here as test anxiety, and academic achievement.

The present study is also informed by Self-Regulated Learning theory (SRL; Zimmerman, 2000), which emphasizes that academic achievement is shaped not only by cognitive ability but by students’ capacity to monitor, regulate, and direct their own learning behaviors. SRL theory is particularly relevant to the distance education context, where reduced external structure places greater demands on students’ self-regulatory resources (Broadbent and Poon, 2015). In this sense, procrastination and perfectionism can be understood not only as anxiety-driven regulatory responses within the SEVT framework, but also as manifestations of self-regulatory difficulty that may be particularly relevant to consider in autonomous, low-scaffolding learning environments. The integration of SEVT and SRL perspectives provides a useful interpretive framework for considering how emotional costs may be associated with self-regulatory processes and academic achievement. The present study does not directly test the core constructs of either theory but interprets the observed associations within this broader theoretical perspective.

Finally, exam format preference is incorporated into the model as a self-reported individual-difference moderator. Although it is not conceptualized as a direct measure of expectancy beliefs, SEVT provides a useful theoretical framework for considering why the associations between anxiety-related self-regulatory processes and academic achievement may differ across students reporting different examination-format preferences. Accordingly, exam format preference is expected to moderate the associations between academic procrastination and academic achievement and between academic perfectionism and academic achievement.

## 3 The significance, aim, and hypothesis of the study

Building on the limitations of direct-effect models, the purpose of this study was to examine the association between test anxiety and academic achievement by considering multiple self-regulatory processes and individual-level contextual factors within an integrated Situated Expectancy-Value (SEVT) and Self-Regulated Learning (SRL) framework.

Within this framework, test anxiety is conceptualized as a perceived cost that may disrupt students’ self-regulatory engagement with academic tasks. Academic procrastination represents a cost-avoidance process, in which heightened anxiety may increase the likelihood of task delay in order to minimize immediate distress. Academic perfectionism represents an overcontrol process in which anxious students may seek to eliminate potential errors through excessive performance monitoring. Both processes are expected to be associated with lower academic achievement: procrastination through its association with limited preparation, and perfectionism through its association with reduced cognitive flexibility and sustained evaluative anxiety during examinations.

**Figure 1:**
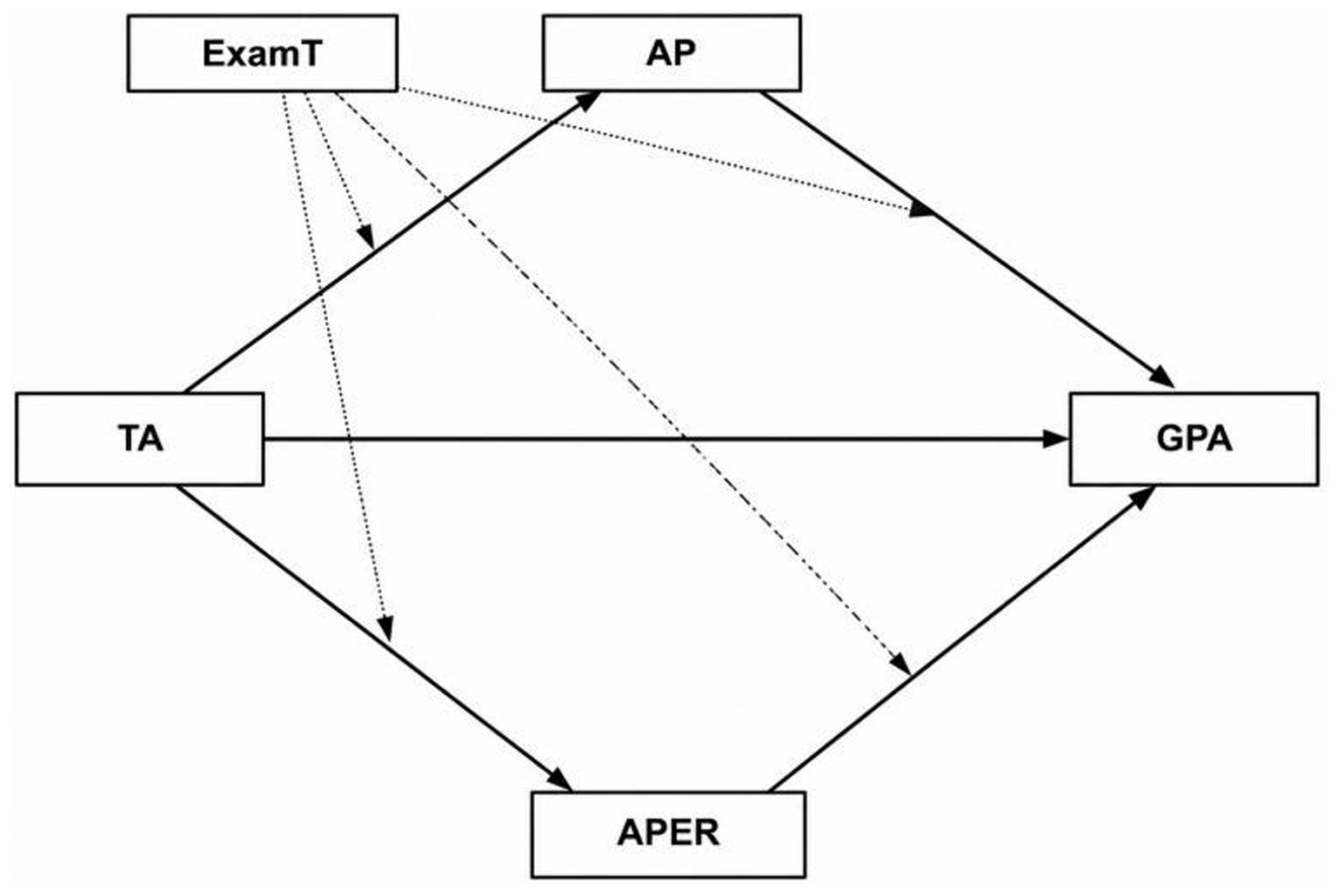
Proposed theoretical model.

These arguments collectively highlight a critical gap in the literature, indicating that the association between test anxiety and academic performance may be better understood by considering multiple self-regulatory processes rather than focusing solely on a direct association. As such, academic procrastination and academic perfectionism are hypothesized to serve as parallel mediators. Furthermore, exam format preference is expected to moderate the associations between academic procrastination and academic achievement and between academic perfectionism and academic achievement. Based on these theoretical considerations, the following hypotheses were formulated (see Figure 1):

*H_1_*: Test anxiety is positively associated with academic procrastination.

*H_2_*: Test anxiety is positively associated with academic perfectionism.

*H_3_*: Academic procrastination is negatively associated with academic achievement.

*H_4_*: Academic perfectionism is negatively associated with academic achievement.

*H_5a_*: Academic procrastination mediates the associations between test anxiety and academic achievement, such that test anxiety is positively associated with academic procrastination, which is in turn negatively associated with academic achievement.

*H_5b_*: Academic perfectionism mediates the associations between test anxiety and academic achievement, such that test anxiety is positively associated with academic perfectionism, which is in turn negatively associated with academic achievement.

*H_6a_*: Exam format preference moderates the indirect association of test anxiety on academic achievement via academic procrastination.

*H_6b_*: Exam format preference moderates the indirect association of test anxiety on academic achievement via academic perfectionism.

## 4 Materials and methods

### 4.1 Participants

The participants of this study were university students enrolled in the Open Education System during the Fall and Spring semesters of the 2024–2025 academic year in Türkiye. Instruction is delivered primarily through asynchronous learning resources, including pre-recorded video lectures provided via the university’s online learning platform, digital textbooks, and supplementary course materials. Real-time synchronous classes are not a mandatory component of the instructional model. Participation was voluntary. The psychological measures (test anxiety, academic procrastination, academic perfectionism, and exam format preference) were administered online through the university’s learning platform before the Spring semester final examination period of the 2024–2025 academic year. Eligible participants were students who were actively enrolled in the Open Education System during the 2024–2025 academic year and who provided informed consent to participate in the study. Because data collection took place before the Spring semester final examinations, all participants had completed at least one semester in the Open Education System and had experienced at least one examination period before completing the questionnaires.

A total of 928 students participated, comprising 592 females (63.8%) and 336 males (36.2%). More than half of the participants (52.7%) were young adults (aged 17–35), 44.8% were middle-aged adults (aged 36–64), and 2.5% were aged 65 or older, representing the late adulthood group. Regarding examination-format preference, 630 students (67.9%) preferred multiple-choice examinations, whereas 298 students (32.1%) preferred constructed-response examinations.

Academic achievement was operationalized as cumulative grade point average (GPA), obtained from official institutional records in accordance with the ethical approval granted for this study and participants’ informed consent. In contrast to the psychological measures, GPA records were retrieved from the university’s official student information system after the conclusion of the Spring semester, once final grades had been officially posted. Consequently, the cumulative GPA reflected students’ academic performance accumulated across all completed semesters up to the end of the 2024–2025 academic year, including grades earned prior to the administration of the psychological measures as well as grades from the Spring semester, which were finalized and recorded after data collection. The cumulative GPA ranged from 0.00 to 4.00 in accordance with the university’s standard grading system. Although grading practices may differ across programmes, all GPA scores were obtained from the university’s official academic records and are based on the same institutional 4-point grading system. All course grades contributing to the cumulative GPA were derived from courses assessed using multiple-choice examinations. The use of institutionally sourced GPA data minimized potential response bias and enhanced the validity of the outcome measure.

The study employed a cross-sectional, correlational design. Test anxiety, academic procrastination, academic perfectionism, and examination-format preference were assessed at a single measurement occasion, prior to the Spring semester final examinations, whereas academic achievement was represented by institutionally recorded cumulative GPA retrieved after the semester’s conclusion. Because the cumulative GPA combined academic performance recorded before the psychological measures with grades finalized afterward, it cannot be treated as a purely prospective outcome measured entirely subsequent to the psychological variables, nor as one determined entirely beforehand. Therefore, the proposed ordering of variables reflects a theoretically specified statistical model tested in this study and should not be interpreted as establishing a fully prospective temporal or causal sequence.

### 4.2 Measures

#### 4.2.1 Demographic information form

A demographic information form was used to collect background information including gender, age, and exam format preference. Students’ preference for examination format was assessed using a single item: “Which exam format do you prefer?” with two response options (1 = multiple-choice, 2 = constructed-response). This item was designed to capture students’ self-reported examination-format preference as an individual-difference variable. It was not intended to directly measure expectancy beliefs, perceived controllability, anticipated success, or other SEVT constructs. It is important to note that all participants completed multiple-choice examinations; therefore, exam format preference is conceptualized as an individual-difference variable that may reflect students’ subjective orientations toward evaluative contexts, rather than a direct indicator of the objective exam format experienced.

#### 4.2.2 Westside test anxiety scale

Developed by Driscoll (2007) and adapted to Turkish by Totan and Yavuz (2009), this one-dimensional 5-point Likert-type scale measures test anxiety. Exploratory factor analysis indicated that the scale explained 46.05% of the total variance, while confirmatory factor analysis showed acceptable model fit (*χ*^2^/df = 3.69, RMSEA = 0.04, CFI = 0.97, GFI = 0.93, NFI = 0.96, RMR = 0.05). Cronbach’s alpha reliability coefficient was 0.89.

#### 4.2.3 Academic Perfectionism Scale

Developed by Odacı et al. (2017), this three-factor (self-doubt, comparison, idealisation) 5-point Likert-type scale measures academic perfectionism. Exploratory factor analysis revealed that the scale explained 52.36% of the variance, and confirmatory factor analysis confirmed an acceptable fit (*χ*^2^/df = 2.36, RMSEA = 0.06, CFI = 0.91, GFI = 0.94, AGFI = 0.91, NNFI = 0.89, SRMR = 0.05). Cronbach’s alpha reliability coefficient was 0.82. In the present study, the scale was used to operationalize maladaptive academic perfectionism (perfectionistic concerns) rather than adaptive perfectionistic strivings. Specifically, its dimensions primarily assess chronic self-doubt, maladaptive social comparison, and rigid or unrealistically high academic standards, which are conceptually consistent with the perfectionistic concerns dimension described in the broader perfectionism literature (Flett and Hewitt, 2002; Frost et al., 1990; Stoeber and Otto, 2006). Consequently, higher scores indicate greater maladaptive perfectionistic tendencies in academic contexts.

#### 4.2.4 Academic procrastination scale-short form

Developed by McCloskey (2011) and adapted into Turkish by Balkıs and Duru (2022), this one-dimensional 5-point Likert-type scale measures academic procrastination. Confirmatory factor analysis demonstrated good model fit (*χ*^2^/df = 1.453, RMSEA = 0.04, SRMR = 0.02, GFI = 0.99, NFI = 0.99, RFI = 0.98), and Cronbach’s alpha reliability coefficient was 0.88.

### 4.3 Data analysis

Data was analyzed using IBM SPSS Statistics (version 26), Jamovi (version 2.4.14), and PROCESS macro. A total of 954 participants completed the survey. The dataset was screened for duplicate and otherwise invalid responses (e.g., multiple submissions by the same individual), resulting in the exclusion of 24 cases. No missing data were identified. The cleaned dataset was then examined for outliers. No univariate outliers were detected. Multivariate outliers were assessed using Mahalanobis distance (*p* < 0.001), and two additional cases were removed. Consequently, the final sample consisted of 928 participants.

The assumptions of normality, linearity, homoscedasticity, and multicollinearity were also checked. A review of the descriptive statistics, which included mean, median, mode, skewness, kurtosis, was used to check for normality. Scatter plots were also created to visually inspect for non-linearity. Additionally, multicollinearity was assessed using several diagnostic measures, which include correlation coefficients, variance inflation factors (VIF), tolerance, and condition indexes. These results did not indicate a problem with multicollinearity (*r* < 0.80, VIF < 10, tolerance > 0.20, condition index < 30; Leech et al., 2005). Common Method Bias (CMB) was assessed using Harman’s Single-Factor Test (Podsakoff et al., 2012). The first factor explained 36.65% of the variance. There was no indication of CMB present.

A two-step analytic approach was adopted in the present study. In the first step, structural equation modeling (SEM) was conducted using Jamovi (Version 2.4.14; Gallucci and Jentschke, 2021) to evaluate the measurement model and to estimate the hypothesized mediation model (Kline, 2015). Before interpreting the structural relationships among the latent variables, the measurement properties of the model were evaluated by examining standardized factor loadings, Cronbach’s alpha (Cα), composite reliability (CR), AVE, and HTMT. Model fit was evaluated using several standard indices, including *χ*^2^/df, Root Mean Square Error of Approximation (RMSEA), Standardized Root Mean Squared Residual (SRMR), Comparative Fit Index (CFI), and Tucker-Lewis Index (TLI), in accordance with Hu and Bentler (1999) and Kline (2015) recommendations for acceptable cutoff criteria. Multivariate normality was assessed using Mardia’s coefficients. The results indicated significant multivariate skewness (coefficient = 59.80, *χ*^2^(4960) = 9244.00, *p* < 0.001) and multivariate kurtosis (coefficient = 1086.80, *z* = 44.10, *p* < 0.001). Confirmatory factor analysis (CFA) was conducted using the robust Maximum Likelihood (MLR) estimator implemented in the SEMLj module of Jamovi. Although the questionnaire items were measured on five-point Likert-type scales and treated as continuous indicators, multivariate normality was violated according to Mardia’s coefficients. Therefore, the MLR estimator was preferred because it provides robust standard errors and a scaled test statistic under non-normality. Academic perfectionism was specified as a second-order latent construct represented by three first-order dimensions (Self-Doubt, Comparison, and Idealization), corresponding to the theoretical structure of the Academic Perfectionism Scale. Based on theoretical and content-related similarity between items, correlated residuals were specified between TA8 and TA9, and TA3 and TA4. No additional *post hoc* model modifications were performed.

In the second step, moderated mediation was tested using Conditional Process Analysis (PROCESS Model 58; Hayes, 2022) implemented in the PROCESS macro for IBM SPSS Statistics (Version 26.0). A complementary two-stage analytical strategy was adopted. In the first stage, structural equation modeling (SEM) was used to evaluate the measurement model and estimate the hypothesized mediation model using latent variables, thereby establishing the adequacy of the measurement properties of the study constructs. In the second stage, moderated mediation was examined using PROCESS Model 58 with observed summed scale scores. This second analysis was conducted to determine whether the indirect associations identified in the mediation model differed according to students’ examination-format preferences. Thus, the SEM and PROCESS analyses served complementary purposes: SEM established the measurement model and tested the mediation model at the latent-variable level, whereas PROCESS evaluated whether these indirect associations varied across examination-format preference groups. In the measurement model, the multidimensional structure of the Academic Perfectionism Scale was confirmed. For the moderated mediation analyses, the overall academic perfectionism score was used as an observed representation of the construct. This approach was consistent with the theoretical focus of the study on the common overcontrol-related characteristics shared across the three dimensions rather than on the unique effects of the individual dimensions. In this study, test anxiety (TA) was the independent variable, academic achievement (GPA) was the dependent variable, academic procrastination (AP) and academic perfectionism (APER) were both parallel mediators, and exam format preference was the moderator. Examination-format preference was coded as 0 = multiple-choice (reference group) and 1 = constructed-response. As a sensitivity analysis, participants’ age (continuous) and programme level (0 = associate degree, 1 = bachelor’s degree) were included as covariates in the moderated mediation analyses to examine the robustness of the findings. Continuous predictors involved in interaction terms were mean-centered prior to analysis, whereas the dichotomous moderator (examination format preference) and programme level retained their original coding. Conditional process models provide a statistical framework for examining theoretically specified indirect associations while assessing whether these associations differ across levels of a moderating variable (Hayes, 2022; Hayes and Rockwood, 2020; Xu et al., 2024). Indirect effects were calculated using 5,000 bootstrap resamples at a 95% confidence interval (Preacher and Hayes, 2008). Graphical representations of the moderation effects were also produced using the PROCESS macro.

## 5 Results

### 5.1 Descriptive statistics and correlations

Descriptive statistics were computed for all variables, and Pearson correlation coefficients were calculated to examine the relationships among TA, AP, APER, and GPA. Table 1 presents the means, standard deviations, and correlation coefficients of the constructs.

**Table 1:** Descriptive statistics and correlations among study variables.

Variables	*M*	SD	Skewness coefficient	Kurtosis coefficient	1	2	3	4
1. TA	31.772	10.749	0.177	−0.714	–			
2. AP	11.845	5.202	0.669	−0.347	0.333*	–		
3. APER	36.709	10.167	0.317	−0.693	0.637*	0.231*	–	
4. GPA	2.141	0.913	−0.161	−0.666	−0.347*	−0.212*	−0.327*	–

TA, Test Anxiety; AP, Academic Procrastination; APER, Academic Perfectionism; GPA, Grade Point Average; *p < 0.001.

Table 1 displays significant relationships among the constructs in the model. Test anxiety was positively correlated with both academic procrastination and academic perfectionism (respectively r_TA-AP_ = 0.333, r_TA-APER_ = 0.637, *p* < 0.05), while all three variables were negatively correlated with GPA (respectively r_TA-GPA_ = −0.347, r_AP-GPA_ = −0.212, r_APER-GPA_ = −0.327, *p* < 0.05).

### 5.2 Results regarding the theoretical model

**Figure 2:**
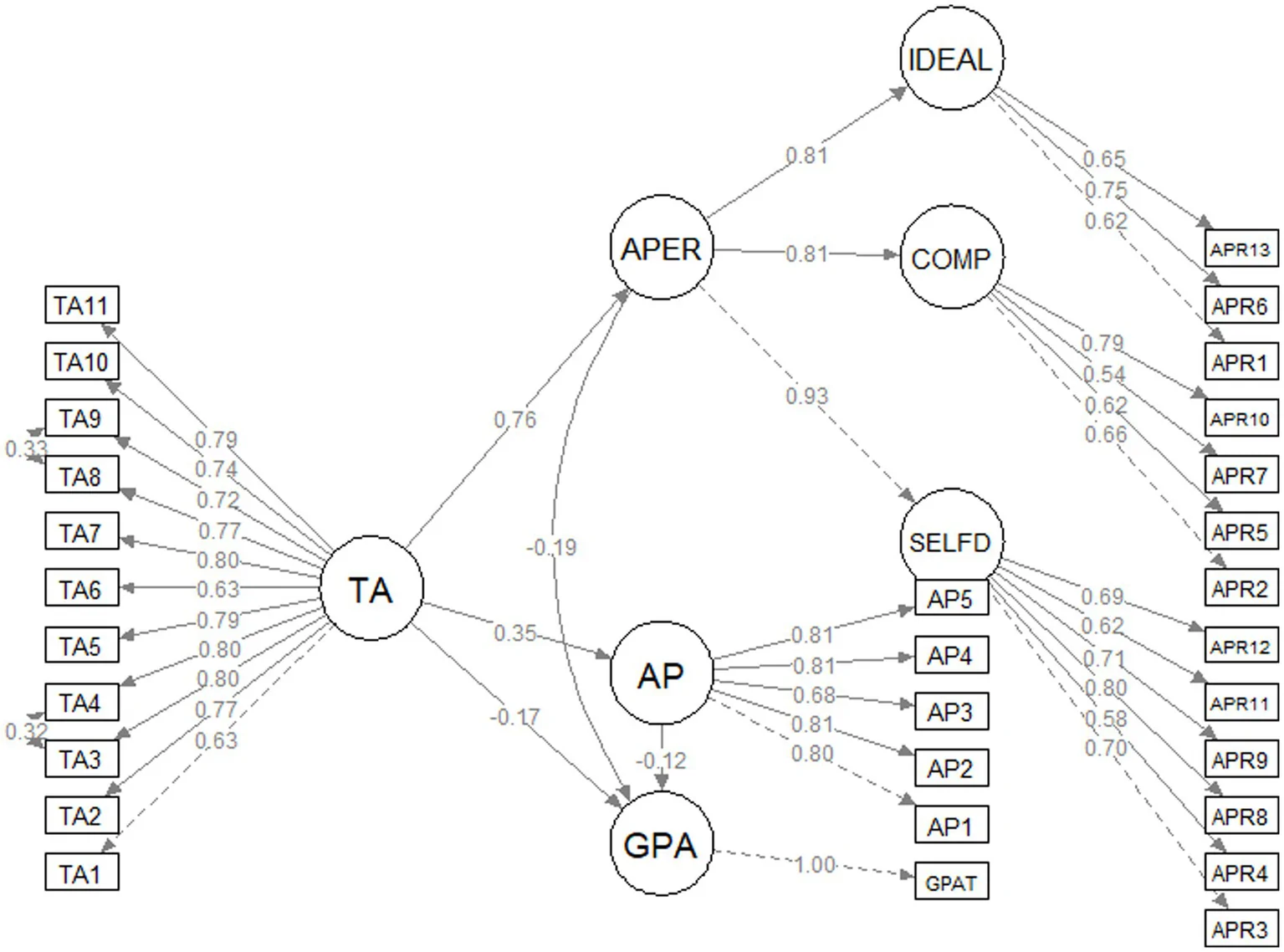
Results of structural model.

First, the reliability and validity of the constructs were established using Cronbach’s alpha (Cα), composite reliability (CR), average variance extracted (AVE), and the heterotrait-monotrait ratio (HTMT). The hypothesized mediation model was then estimated, and the standardized factor loadings and model fit indices reported below were derived from the final mediation model. Standardized factor loadings ranged from 0.536 to 0.815 (see Figure 2). All variables’ factor loadings were greater than 0.50 (Chin, 2009). Cronbach’s alpha (Cα) and composite reliability (CR) were examined to assess internal consistency reliability, AVE was used to assess convergent validity, and HTMT was used to assess discriminant validity. Specifically, test anxiety (TA) showed high reliability (Cα = 0.934; CR = 0.934) and adequate convergent validity (AVE = 0.564). Academic procrastination (AP) also demonstrated acceptable reliability (Cα = 0.887; CR = 0.889) and adequate convergent validity (AVE = 0.613). Academic perfectionism (APER) exhibited good reliability (Cα = 0.886; CR = 0.916). Although the AVE values for academic perfectionism (0.458) fell marginally below the conventionally recommended threshold of 0.50 (Fornell and Larcker, 1981), its standardized factor loadings were all above 0.50 and its high composite reliability (CR = 0.916) indicates acceptable convergent validity. Convergent validity may still be considered acceptable when composite reliability exceeds 0.70 despite an AVE value below 0.50 (Fornell and Larcker, 1981; Hair et al., 2010). Prior research has noted that AVE is a conservative criterion, and that adequate reliability combined with theoretically grounded factor structures may compensate for marginally subthreshold AVE values (Hair et al., 2010). Furthermore, discriminant validity was supported, as all HTMT values were below the recommended threshold of 0.85 (Henseler et al., 2015). Specifically, the HTMT values were 0.354 between test anxiety (TA) and academic procrastination (AP), 0.690 between test anxiety (TA) and academic perfectionism (APER), and 0.227 between academic procrastination (AP) and academic perfectionism (APER), indicating adequate discriminant validity among the constructs. The goodness-of-fit indices for the model were within acceptable ranges [*χ*^2^/df = 3.86 (1,529/396), robust RMSEA = 0.060, SRMR = 0.055, robust CFI = 0.913, RNI = 0.913, TLI = 0.904], confirming the overall adequacy of the model fit.

Table 2 presents the results of the path analysis examining the mediating effects of the variables. The direct effect of test anxiety (TA) on GPA was found to be negative and significant in the absence of mediators [Estimate = −0.402, 95% CI (−0.478, −0.327), *β* = −0.352, *p* < 0.001]. Positive and significant relationships were observed between TA and both academic procrastination [AP; Estimate = 0.462, 95% CI (0.360, 0.564), *β* = 0.350, *p* < 0.001] and academic perfectionism [APER; Estimate = 0.706, 95% CI (0.593, 0.820), *β* = 0.761, *p* < 0.001], supporting H_1_ and H_2_. After the mediators were included in the model, TA remained negatively associated with GPA [Estimate = −0.190, 95% CI (−0.328, −0.053), *β* = −0.165, *p* = 0.007]. In addition, both AP [Estimate = −0.102, 95% CI (−0.164, −0.039), *β* = −0.117, *p* = 0.001] and APER [Estimate = −0.239, 95% CI (−0.395, −0.082), *β* = −0.192, *p* = 0.003] were negatively associated with GPA, thereby supporting H_3_ and H_4_. The indirect effects of TA on GPA through AP [Estimate = −0.047, 95% CI (−0.077, −0.017), *β* = −0.041, *p* = 0.002] and APER [Estimate = −0.169, 95% CI (−0.276, −0.061), *β* = −0.146, *p* = 0.002] were both statistically significant. Because the direct association between TA and GPA remained significant after the mediators were included in the model, the findings indicate partial mediation; therefore, H_5a_ and H_5b_ were supported.

**Table 2:** Results of path analysis on the mediation of AP and APER.

Path coefficients and indirect effects	Estimate	SE	*β*	*z*	*p*	95% CI
TA ⇒ AP	0.462	0.052	0.350	8.860	<0.001	[0.360, 0.564]
TA ⇒ APER	0.706	0.058	0.761	12.210	<0.001	[0.593, 0.820]
TA ⇒ GPA	−0.190	0.070	−0.165	−2.710	0.007	[−0.328, −0.053]
AP ⇒ GPA	−0.102	0.032	−0.117	−3.190	0.001	[−0.164, −0.039]
APER ⇒ GPA	−0.239	0.080	−0.192	−3.000	0.003	[−0.395, −0.082]
TA ⇒ AP ⇒ GPA	−0.047	0.015	−0.041	−3.083	0.002	[−0.077, −0.017]
TA ⇒ APER ⇒ GPA	−0.169	0.055	−0.146	−3.074	0.002	[−0.276, −0.061]

TA, Test Anxiety; AP, Academic Procrastination; APER, Academic Perfectionism; GPA, Grade Point Average; CI, 95% confidence interval. Estimates and 95% confidence intervals represent unstandardized coefficients, whereas *β* values represent standardized estimates.

To examine whether examination-format preference moderated the associations between test anxiety, the proposed self-regulatory processes, and academic achievement, a moderation analysis was conducted using PROCESS Model 58. Specifically, examination-format preference was specified as the moderator of the associations between test anxiety and academic procrastination, test anxiety and academic perfectionism, academic procrastination and academic achievement, and academic perfectionism and academic achievement. The results of these interaction analyses are presented in Table 3.

**Table 3:** Interaction effects for the moderation analysis.

Interaction	Estimate	SE	*t*	*p*	95% CI
TAxEFP ⇒ AP	−0.051	0.032	−1.604	0.109	[−0.114, 0.011]
TAxEFP ⇒ APER	−0.050	0.051	−0.973	0.331	[−0.150, 0.051]
APxEFP ⇒ GPA	0.025	0.011	2.216	0.027	[0.003, 0.048]
APERxEFP ⇒ GPA	−0.004	0.006	−0.606	0.545	[−0.015, 0.008]

TA, Test Anxiety; AP, Academic Procrastination; APER, Academic Perfectionism; GPA, Grade Point Average; EFP, Exam Format Preference; CI, 95% bias-corrected bootstrap confidence intervals. Estimates are unstandardized coefficients.

The moderation analyses indicated that the model explained 11.7% of the variance in academic procrastination [AP; *R*^2^ = 0.117, *F*(3, 924) = 40.634, *p* < 0.001], 40.6% of the variance in academic perfectionism [APER; *R*^2^ = 0.406, *F*(3, 924) = 210.598, *p* < 0.001], and 15.8% of the variance in academic achievement [GPA, *R*^2^ = 0.158, *F*(6, 921) = 28.823, *p* < 0.001]. As shown in Table 3, examination format preference did not significantly moderate the association between TA and AP [Estimate = −0.051, SE = 0.032, *t* = −1.604, *p* = 0.109, 95% CI (−0.114, 0.011), Δ*R*^2^ = 0.002], nor did it moderate the association between TA and APER [Estimate = −0.050, SE = 0.051, *t* = −0.973, *p* = 0.331, 95% CI (−0.150, 0.051), Δ*R*^2^ = 0.001]. However, examination format preference significantly moderated the association between AP and GPA [Estimate = 0.025, SE = 0.011, *t* = 2.216, *p* = 0.027, 95% CI (0.003, 0.048), Δ*R*^2^ = 0.004]. In contrast, the interaction between APER and examination format preference was not statistically significant [Estimate = −0.004, SE = 0.006, *t* = −0.606, *p* = 0.545, 95% CI (−0.015, 0.008), Δ*R*^2^ < 0.001]. These findings indicate that examination format preference moderated only the association between academic procrastination and academic achievement.

**Figure 3:**
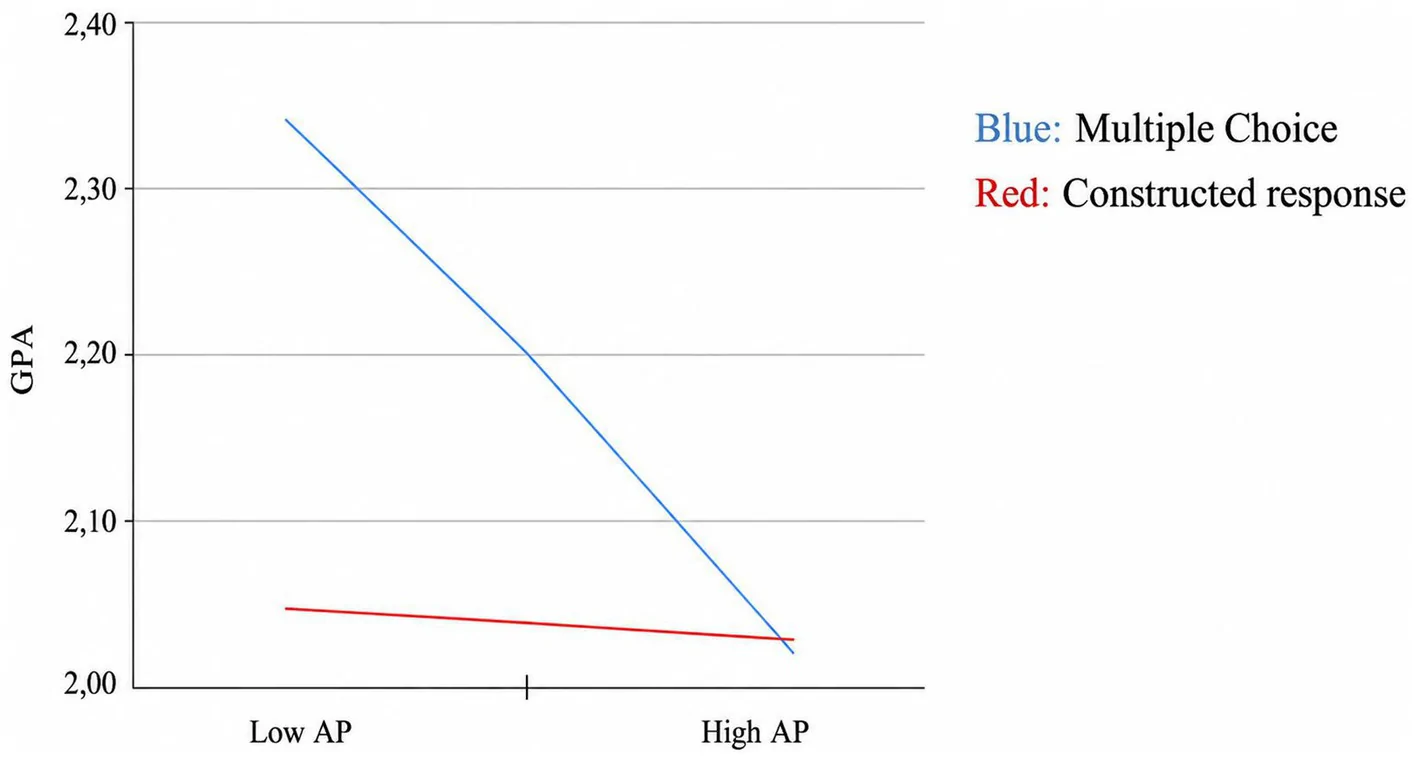
Exam format preference is a moderator of the relationship between AP and GPA.

The moderated mediation analysis (Model 58) examined whether the indirect associations between test anxiety and academic achievement differed according to examination-format preference. As shown in Table 4, the conditional indirect effect of test anxiety (TA) on academic achievement (GPA) through academic procrastination (AP) was significant for students who preferred multiple-choice examinations [Effect = −0.005, BootSE = 0.001, 95% BC CI (−0.008, −0.002)], whereas the corresponding conditional indirect effect was not significant for students who preferred constructed-response examinations [Effect = 0.000, BootSE = 0.001, 95% BC CI (−0.002, 0.002)]. To formally evaluate moderated mediation, the index of moderated mediation and its bootstrap confidence interval were examined. For the indirect association through AP, the index of moderated mediation was statistically significant [Index = 0.005, BootSE = 0.002, 95% BC CI (0.001, 0.008)], indicating that the conditional indirect association through AP differed significantly between the two examination-format preference groups. The conditional indirect effects of TA on GPA through APER were statistically significant in both examination-format preference groups (see Table 4). However, the index of moderated mediation was not statistically significant [Index = −0.001, BootSE = 0.004, 95% BC CI (−0.009, 0.006)], indicating that although the indirect effect through APER was statistically significant in both groups, it did not differ significantly according to examination-format preference. Taken together, these findings support H_6a_ but not H_6b_. Specifically, the conditional indirect association through academic procrastination differed significantly according to examination-format preference, whereas the conditional indirect association through academic perfectionism was statistically significant in both groups but did not differ significantly between them. As shown in Figure 3, the association between AP and GPA varied according to students’ self-reported examination-format preference, with higher levels of academic procrastination being associated with lower GPA scores among students who preferred multiple-choice examinations but not among those who preferred constructed-response examinations.

**Table 4:** Conditional indirect effects and indices of moderated mediation.

Exam format preference	Indirect association	Effect	BootSE	95% CI
Multiple choice	TA ⇒ AP ⇒ GPA	−0.005	0.001	[−0.008, −0.002]
	TA ⇒ APER⇒ GPA	−0.009	0.003	[−0.014, −0.004]
Constructed response	TA ⇒ AP ⇒ GPA	0.000	0.001	[−0.002, 0.002]
	TA ⇒ APER⇒ GPA	−0.011	0.003	[−0.017, −0.005]

Index of moderated mediation	Index	BootSE	95% CI
TA ⇒ AP ⇒ GPA	0.005	0.002	[0.001, 0.008]
TA ⇒ APER⇒ GPA	−0.001	0.004	[−0.009, 0.006]

TA, Test Anxiety; AP, Academic Procrastination; APER, Academic Perfectionism; GPA, Grade Point Average; CI, 95% bias-corrected bootstrap confidence intervals.

As a sensitivity analysis, the moderated mediation model was re-estimated while controlling for participants’ age and programme level. Age was negatively associated with academic procrastination (*B* = −0.063, SE = 0.013, *p* < 0.001) and negatively associated with academic perfectionism (*B* = −0.092, SE = 0.021, *p* < 0.001), and showed a marginal association with GPA (*B* = 0.005, SE = 0.002, *p* = 0.051). Programme level showed a marginal association with academic procrastination (*B* = 0.644, SE = 0.329, *p* = 0.051) and was positively associated with GPA (*B* = 0.134, SE = 0.057, *p* = 0.019). Importantly, controlling for these covariates did not alter the pattern of results. All previously significant direct, interaction, and conditional indirect effects remained statistically significant, whereas all previously non-significant effects remained non-significant, indicating that the moderated mediation findings were robust to adjustment for age and programme level.

## 6 Discussion

This study offers a process-based perspective on the association between test anxiety and academic achievement by incorporating both avoidance-based and overcontrol-based self-regulatory processes within an integrated theoretical framework. Consistent with prior research, test anxiety was positively associated with both academic procrastination and academic perfectionism, suggesting that students experiencing evaluative anxiety engage in two qualitatively distinct forms of self-regulatory response: disengagement through procrastination, aimed at reducing immediate distress (Sirois and Pychyl, 2013), and overcontrol through perfectionism, driven by fear of making mistakes and heightened performance pressure (Flett and Hewitt, 2002; Stoeber and Otto, 2006). Together, these findings indicate that procrastination and perfectionism represent complementary self-regulatory constructs that are statistically associated with the indirect association between test anxiety and academic achievement in the present model.

Academic procrastination and academic perfectionism were both found to be inversely related to academic achievement. These findings are consistent with prior meta-analysis demonstrating a negative association between procrastination and academic achievement, together with lower academic engagement and reduced preparation (Steel, 2007; Kim and Seo, 2015). Academic perfectionism has been previously related to increased concern regarding evaluation and impaired performance during high-stakes situations (Flett and Hewitt, 2002; Núñez-Peña and Bono, 2021; Stoeber and Otto, 2006). Although prior research has demonstrated that both avoidance and overcontrol are associated with poorer academic performance in high-stakes academic settings (Flett and Hewitt, 2002; Steel, 2007), the present findings extend this literature by suggesting that both avoidance-based and overcontrol-based self-regulatory processes are theoretically relevant to the association between test anxiety and academic achievement.

More importantly, the mediation results are consistent with the possibility that the association between test anxiety and academic achievement is statistically related, in part, to behavioral regulatory processes. Rather than being reflected solely in cognitive interference during examinations (Eysenck et al., 2007), test anxiety also appears to be associated with students’ preparatory behaviors before evaluation, highlighting the importance of considering self-regulatory processes beyond the testing moment itself. These findings are consistent with the broader empirical literature documenting associations between test anxiety, self-regulation, and other psychological correlates of academic performance (von der Embse et al., 2018). In line with self-regulated learning theory (Zimmerman, 2000), these findings suggest that, in distance education contexts where external regulatory scaffolding is limited, behavioral regulation may be an important correlate of academic outcomes. Specifically, anxiety-related behaviors such as procrastination and perfectionism may reflect different forms of self-regulatory difficulty across phases of the learning process. The differential moderating role of exam format preference further suggests that self-regulatory processes are not uniform across evaluative contexts but may vary according to students’ self-reported examination-format preferences.

The moderating results demonstrate the association between academic procrastination and academic achievement differed according to students’ self-reported examination-format preferences. Within the SEVT framework (Eccles and Wigfield, 2020), these differences may be interpreted cautiously. Students’ self-reported exam-format preferences may reflect a range of prior experiences, subjective impressions, or motivational orientations toward evaluative situations, but they were not assessed as direct indicators of expectancy beliefs or other SEVT constructs. However, because all participants completed multiple-choice examinations and examination format was not experimentally manipulated, these interpretations remain speculative and should not be interpreted as evidence of the effects of examination format itself. Accordingly, the findings are best understood as reflecting differences associated with students’ self-reported examination-format preferences rather than causal effects of examination format per se.

It is also important to consider the magnitude of the observed moderation effect alongside its statistical significance. Although exam format preference significantly moderated the association between academic procrastination and GPA, the interaction effect was small (Δ*R*^2^ = 0.004). Likewise, the index of moderated mediation indicated a statistically reliable but small conditional indirect effect [Index = 0.005, 95% BC CI (0.001, 0.008)]. Given the large sample size (*N* = 928), even small effects can reach conventional levels of statistical significance; therefore, statistical significance in this context should not be equated with strong or practically substantial moderation. Rather, the finding is best interpreted as indicating a reliable but relatively small differentiation in how academic procrastination relates to GPA across students with different examination-format preferences. This distinction is consistent with broader methodological guidance suggesting that interaction effects in field research involving observed (non-experimental) individual-difference moderators are typically small in magnitude (Aguinis et al., 2005; McClelland and Judd, 1993), and that their theoretical and practical importance should be evaluated independently of statistical significance alone. Accordingly, while the present findings support the theoretical proposition that avoidance-based self-regulatory processes are more sensitive to exam format preference than overcontrol-based processes, the practical implications of this differentiation, such as instructional design or assessment policy, should be interpreted cautiously and warrant further replication before being translated into applied recommendations.

Exam format preference may represent one individual-difference characteristic that is relevant when interpreting variation in evaluative experiences within the broader SEVT framework. One tentative possibility is that, among students who preferred multiple-choice formats, procrastination was associated withgreater decrements in academic achievement because multiple-choice examinations may rely more heavily on prior preparation. However, because exam format preference was assessed as a self-reported preference rather than as a direct measure of students’ perceptions, expectations, or evaluative concerns, this explanation remains speculative and should be interpreted cautiously. Conversely, the indirect association through perfectionism was not moderated by exam format preference, suggesting that overcontrol-based processes may be relatively stable across students with different examination-format preferences compared to avoidance-based processes (Flett and Hewitt, 2002; Stoeber and Otto, 2006). This finding is consistent with the theorized trait-like stability of perfectionistic concerns across evaluative contexts.

Theoretically, the findings are broadly consistent with key assumptions of Situated Expectancy-Value Theory (Eccles and Wigfield, 2002; Eccles and Wigfield, 2020), particularly the role of perceived costs in shaping behavioral responses. In the present study, SEVT is interpreted as an explanatory lens rather than a fully tested model. Test anxiety, conceptualized as a perceived cost, appears to be associated with both avoidance-based and overcontrol-based regulatory responses. Furthermore, these self-regulatory processes may vary according to students’ self-reported examination-format preferences. From an SEVT perspective, such preferences may be associated with individual differences relevant to evaluative situations, although they should not be interpreted as direct measures of expectancy beliefs or other core SEVT constructs. Consistent with this interpretation, the moderating role of exam format preference suggests that the associations between these self-regulatory processes and academic achievement may differ across students reporting different examination-format preferences. Accordingly, the present findings should be interpreted as broadly consistent with SEVT rather than as providing a direct empirical test of an integrated SEVT-SRL model, as the study did not directly assess the theory’s core motivational constructs.

These findings are consistent with recent evidence suggesting that the indirect association between test anxiety and academic achievement through avoidance-based self-regulatory processes may depend on contextual characteristics. Using a conceptually related moderated mediation framework, Aydemir (2026) reported that the indirect association between test anxiety and GPA through academic procrastination differed according to the examination environment among distance learners. Although the moderator examined in that study (exam environment) differs from the self-reported exam format preference investigated here, both studies converge in indicating that the associations between avoidance-based self-regulatory processes, test anxiety, and academic achievement may be context-dependent rather than uniform. Because the studies differ in sample characteristics, moderator operationalization, and disability status assessment, these similarities should be interpreted cautiously and should not be taken as evidence of a common underlying pattern of association.

The present findings are relevant to distance education, where students rely more heavily on self-regulatory processes because of the reduced external structure characteristic of online learning environments (Zimmerman, 2000). Within the SRL framework, academic procrastination and academic perfectionism represent distinct forms of self-regulatory difficulty that may undermine academic achievement when students are required to regulate their own learning with limited external guidance. Accordingly, the present findings are consistent with theoretical accounts emphasizing the importance of self-regulation in distance education. Nevertheless, because the study was conducted exclusively with distance education students and did not compare different instructional contexts or directly assess features such as instructional scaffolding, online support, or behavioral engagement, it cannot be concluded that these associations are unique to, or stronger in, distance education. Rather, the findings suggest that anxiety-related self-regulatory processes may represent one correlate of the variation in academic achievement observed among students in distance education settings.

Practically, the findings have several implications. Interventions targeting test anxiety should not focus only on regulating anxiety during examinations but also on regulating students’ preparatory behaviors. Therefore, interventions may be designed to reduce academic procrastination and encourage adaptive self-regulation strategies. Additionally, interventions may be developed to reduce academic perfectionism, helping students to establish more flexible performance standards and reduce excessive evaluative concerns. Finally, the findings suggest that students’ self-reported examination-format preferences may be relevant when designing future research and when considering individual differences in assessment experiences. However, the present results do not justify redesigning examinations on the basis of students’ preferences alone. Experimental studies manipulating examination format are needed before such recommendations can be made.

Although this study makes significant contributions, there are several limitations. First, although the psychological measures were administered before the spring semester final examinations, academic achievement was operationalized as cumulative GPA, which reflected students’ academic performance accumulated across all completed semesters, including grades earned prior to the psychological measures administration. Consequently, the temporal ordering assumed in the statistical model cannot be established, and the estimated indirect associations should be interpreted as cross-sectional, theory-driven associations rather than evidence of temporal or causal relationships. This limitation is particularly relevant in light of recent longitudinal research demonstrating that the relationships among self-efficacy, expectancy beliefs, test anxiety, and academic performance unfold across multiple measurement occasions rather than at a single point in time (Roick and Ringeisen, 2017). Accordingly, the present findings should be interpreted as identifying theoretically plausible self-regulatory associations rather than establishing the temporal sequence through which these associations may unfold. Reverse or reciprocal associations among the study variables are also possible. In addition, although the present study specified academic perfectionism as a mediator within the theoretically specified model, alternative conceptualizations are also plausible. Academic perfectionism may precede test anxiety, moderate its association with academic achievement, or demonstrate reciprocal associations with test anxiety. Because the present study employed a cross-sectional design, these competing theoretical models cannot be empirically distinguished and should be examined in future longitudinal research. Second, the use of self-report measures for the psychological variables may have introduced common method variance. Moreover, although self-report measures capture students’ subjective perceptions, they cannot directly assess observable engagement within online learning environments. Recent research using Learning Management System (LMS) interaction data has shown that anxiety-related disengagement can also be identified through objective behavioral indicators rather than self-report alone (Taşkın and Kokoç, 2025). Future studies should therefore integrate psychological self-report measures with digital trace data to determine whether perceived procrastination corresponds to objectively observable reductions in online behavioral engagement. Third, exam format preference was measured as a self-reported individual-difference characteristic rather than manipulated experimentally. Therefore, the moderated indirect effects proposed in H_6a_ and H_6b_ should be interpreted as associations contingent on students’ subjective exam format preferences, rather than as evidence that different exam formats causally alter the indirect relationships between test anxiety, academic procrastination/perfectionism, and academic achievement.

Future research should employ longitudinal or experimental designs with temporally separated assessments to evaluate the proposed theoretical ordering of the variables. In addition, future studies should directly assess the core constructs of Situated Expectancy-Value Theory (e.g., expectancy beliefs, task value, and perceived costs) together with validated measures of self-regulated learning to provide a more rigorous test of the proposed theoretical framework. Future research should also examine contextual factors, such as examination difficulty, feedback practices, and characteristics of the online learning environment, to provide a more comprehensive understanding of the associations among test anxiety, self-regulation, and academic achievement. Finally, experimental studies manipulating examination format are needed to determine whether the observed associations reflect students’ self-reported examination-format preferences or characteristics of the assessment format itself.

Overall, the present study offers a broader perspective on the association between test anxiety and academic achievement by considering self-regulatory processes within an integrated theoretical framework. The findings suggest that test anxiety is associated with academic achievement not only through cognitive interference during examinations but also through its associations with students’ preparatory behaviors, reflected in both academic procrastination and academic perfectionism. By incorporating exam format preference as an individual-difference variable, the study further suggests that these associations may differ according to students’ preferences for examination formats. Taken together, the findings provide a more nuanced understanding of how emotional, behavioral, and individual-level factors are jointly associated with academic achievement in distance education settings. Combining longitudinal designs with objective behavioral indicators derived from online learning environments would further clarify both the temporal development and the behavioral manifestation of anxiety-related self-regulatory processes.

## Data Availability

The datasets generated and analysed during the current study are not publicly available due to ethical and privacy restrictions related to participant confidentiality, but they are available from the corresponding author on reasonable request. Requests to access the datasets should be directed to munevver.kaya@marmara.edu.tr.

## References

[ref1] Aguinis H. Beaty J. C. Boik R. J. Pierce C. A. (2005). Effect size and power in assessing moderating effects of categorical variables using multiple regression: a 30-year review. J. Appl. Psychol. 90, 94–107. doi: 10.1037/0021-9010.90.1.94, 15641892

[ref2] Aydemir M. (2026). Online exams buffer the anxiety-procrastination-achievement pathway for distance learners with disabilities. Front. Psychol. 17:1806190. doi: 10.3389/fpsyg.2026.1806190, 42220408 PMC13219380

[ref3] Balkıs M. Duru E. (2022). The examining psychometric characteristics of academic procrastination scale-short form. Pamukkale Univ. J. Educ. 54, 410–425. doi: 10.9779/pauefd.952291

[ref4] Bridgeman B. (1992). A comparison of quantitative questions in open-ended and multiple-choice formats. J. Educ. Meas. 29, 253–271. doi: 10.1111/j.1745-3984.1992.tb00377.x

[ref5] Broadbent J. Poon W. L. (2015). Self-regulated learning strategies and academic achievement in online higher education learning environments: a systematic review. Internet High. Educ. 27, 1–13. doi: 10.1016/j.iheduc.2015.04.007

[ref6] Cassady J. C. Johnson R. E. (2002). Cognitive test anxiety and academic performance. Contemp. Educ. Psychol. 27, 270–295. doi: 10.1006/ceps.2001.1094

[ref7] Chin W. W. (2009). “How to write up and report PLS analyses,” in Handbook of Partial Least Squares: Concepts, Methods and Applications, eds. Vinzi V. E. Chin W. W. Henseler J. Wang H. (Berlin: Springer), 655–690.

[ref8] Chung Y. Shin J. Y. (2024). Profiles of perfectionism and their relations to task disengagement, test anxiety, and depression in south Korean high school students: the mediating role of achievement goals. Eur. J. Educ. 60:e12894. doi: 10.1111/ejed.12894

[ref9] Collignon S. E. Chacko J. Nazir S. (2024). Multiple-choice test format and student test anxiety: a case set in a technical analytics class. J. Inf. Syst. Educ. 35, 467–480. doi: 10.62273/QDMI3914

[ref10] Driscoll R. (2007) Westside test anxiety scale validation. Report No. ED495968. Available online at: https://files.eric.ed.gov/fulltext/ED495968.pdf (Accessed September 15, 2024).

[ref11] Eccles J. S. Adler T. F. Futterman R. Goff S. B. Kaczala C. M. Meece J. L. . (1983). “Expectancies, values, and academic behaviors,” in Achievement and Achievement Motivation, ed. Spence J. T. (San Francisco, CA: W. H. Freeman), 75–146.

[ref12] Eccles J. S. Wigfield A. (2002). Motivational beliefs, values, and goals. Annu. Rev. Psychol. 53, 109–132. doi: 10.1146/annurev.psych.53.100901.135153, 11752481

[ref13] Eccles J. S. Wigfield A. (2020). From expectancy-value theory to situated expectancy-value theory: a developmental, social cognitive, and sociocultural perspective on motivation. Contemp. Educ. Psychol. 61:101859. doi: 10.1016/j.cedpsych.2020.101859

[ref14] Eysenck M. W. Derakshan N. Santos R. Calvo M. G. (2007). Anxiety and cognitive performance: attentional control theory. Emotion 7, 336–353. doi: 10.1037/1528-3542.7.2.336, 17516812

[ref15] Fabian K. Smith S. Taylor-Smith E. Meharg D. (2022). Identifying factors influencing study skills engagement and participation for online learners in higher education during COVID-19. Br. J. Educ. Technol. 53, 1915–1936. doi: 10.1111/bjet.13221, 35601603 PMC9111852

[ref16] Flett G. L. Hewitt P. L. (2002). “Perfectionism and maladjustment: an overview of theoretical, definitional, and treatment issues,” in Perfectionism: Theory, Research, and Treatment, eds. Flett G. L. Hewitt P. L. (Washington, DC: American Psychological Association), 5–31.

[ref17] Fornell C. Larcker D. F. (1981). Evaluating structural equation models with unobservable variables and measurement error. J. Mark. Res. 18, 39–50. doi: 10.2307/3151312

[ref18] Frost R. O. Marten P. Lahart C. Rosenblate R. (1990). The dimensions of perfectionism. Cogn. Ther. Res. 14, 449–468. doi: 10.1007/BF01172967

[ref19] Gallucci M. Jentschke S. (2021) SEMLj: jamovi SEM Analysis [jamovi module]. Available online at: https://semlj.github.io/ (Accessed July 20, 2025).

[ref20] Gopal R. Singh V. Aggarwal A. (2021). Impact of online classes on the satisfaction and performance of students during the pandemic period of COVID 19. Educ. Inf. Technol. 26, 6923–6947. doi: 10.1007/s10639-021-10523-1, 33903795 PMC8059127

[ref21] Hair J. F. Black W. C. Babin B. J. Anderson R. E. (2010). Multivariate Data Analysis. 7th Edn Upper Saddle River, NJ: Pearson.

[ref22] Hayes A. F. (2022). Introduction to Mediation, Moderation, and Conditional Process Analysis: a Regression-Based Approach. 3rd Edn New York, NY: Guilford Publications.

[ref23] Hayes A. F. Rockwood N. J. (2020). Conditional process analysis: concepts, computation, and advances in the modeling of the contingencies of mechanisms. Am. Behav. Sci. 64, 19–54. doi: 10.1177/0002764219859633

[ref24] Henseler J. Ringle C. M. Sarstedt M. (2015). A new criterion for assessing discriminant validity in variance-based structural equation modeling. J. Acad. Mark. Sci. 43, 115–135. doi: 10.1007/s11747-014-0403-8

[ref25] Hu L. Bentler P. (1999). Cutoff criteria for fit indexes in covariance structure analysis: conventional criteria versus new alternatives. Struct. Equ. Model. 6, 1–55. doi: 10.1080/10705519909540118

[ref26] Kim K. R. Seo E. H. (2015). The relationship between procrastination and academic performance: a meta-analysis. Pers. Individ. Dif. 82, 26–33. doi: 10.1016/j.paid.2015.02.038

[ref27] Kline R. B. (2015). Principles and Practices of Structural Equation Modeling. 4th Edn New York, NY: Guilford Press.

[ref28] Leech N. L. Barrett K. C. Morgan G. A. (2005). SPSS for Intermediate Statistics, Use and Interpretation. 2nd Edn Mahwah, NJ: Lawrence Erlbaum Associates Inc.

[ref29] McClelland G. H. Judd C. M. (1993). Statistical difficulties of detecting interactions and moderator effects. Psychol. Bull. 114, 376–390. doi: 10.1037/0033-2909.114.2.376, 8416037

[ref30] McCloskey J. D. (2011). Finally, My Thesis on Academic Procrastination [Master's Thesis]. Arlington (TX): The University of Texas at Arlington.

[ref31] Núñez-Peña M. I. Bono R. (2021). Math anxiety and perfectionistic concerns in multiple-choice assessment. Assess. Eval. High. Educ. 46, 865–878. doi: 10.1080/02602938.2020.1836120

[ref32] Odacı H. Kalkan M. Çikrıkci Ö. (2017). Development of the academic perfectionism scale. Ahi Evran Univ. J. Kırşehir Educ. Fac., 18, 353–366. Available online at https://izlik.org/JA98DD67GE (Accessed September 20, 2024).

[ref33] Onwuegbuzie A. J. (2004). Academic procrastination and statistics anxiety. Assess. Eval. High. Educ. 29, 3–19. doi: 10.1080/0260293042000160384

[ref34] Podsakoff P. M. MacKenzie S. B. Podsakoff N. P. (2012). Sources of method bias in social science research and recommendations on how to control it. Annu. Rev. Psychol. 63, 539–569. doi: 10.1146/annurev-psych-120710-100452, 21838546

[ref35] Preacher K. J. Hayes A. F. (2008). Asymptotic and resampling strategies for assessing and comparing indirect effects in multiple mediator models. Behav. Res. Methods 40, 879–891. doi: 10.3758/brm.40.3.879, 18697684

[ref36] Rodriguez M. C. (2003). Construct equivalence of multiple-choice and constructed-response items: a random effects synthesis of correlations. J. Educ. Meas. 40, 163–184. doi: 10.1111/j.1745-3984.2003.tb01102.x

[ref37] Roick J. Ringeisen T. (2017). Self-efficacy, test anxiety, and academic success: a longitudinal validation. Int. J. Educ. Res. 83, 84–93. doi: 10.1016/j.ijer.2016.12.006

[ref38] Sirois F. M. Pychyl T. A. (2013). Procrastination and the priority of short-term mood regulation: consequences for future self. Soc. Personal. Psychol. Compass 7, 115–127. doi: 10.1111/spc3.12011

[ref39] Steel P. (2007). The nature of procrastination: a meta-analytic and theoretical review of quintessential self-regulatory failure. Psychol. Bull. 133, 65–94. doi: 10.1037/0033-2909.133.1.65, 17201571

[ref40] Stoeber J. Otto K. (2006). Positive conceptions of perfectionism: approaches, evidence, challenges. Personal. Soc. Psychol. Rev. 10, 295–319. doi: 10.1207/s15327957pspr1004_2, 17201590

[ref41] Taşkın N. Kokoç M. (2025). Unveiling the link between test anxiety, online engagement, and academic achievement: insights from LMS interaction data. Interact. Learn. Environ. 33, 2374–2389. doi: 10.1080/10494820.2024.2409406

[ref42] Thomas C. L. Cassady J. C. Heller M. L. (2017). The influence of emotional intelligence, cognitive test anxiety, and coping strategies on undergraduate academic performance. Learn. Individ. Differ. 55, 40–48. doi: 10.1016/j.lindif.2017.03.001

[ref43] Tice D. M. Baumeister R. F. (1997). Longitudinal study of procrastination, performance, stress, and health: the costs and benefits of dawdling. Psychol. Sci. 8, 454–458. doi: 10.1111/j.1467-9280.1997.tb00460.x

[ref44] Totan T. Yavuz Y. (2009). The validity and reliability study of the Turkish version of Westside test anxiety scale. J. Mehmet Akif Ersoy Univ. Fac. Educ. 9, 95–109. Available online at: https://tr-scales.arabpsychology.com/wp-content/uploads/pdf/westside-sinav-kaygisi-olcegi-toad.pdf (Accessed September 18, 2024).

[ref45] von der Embse N. Jester D. Roy D. Post J. (2018). Test anxiety effects, predictors, and correlates: a 30-year meta-analytic review. J. Affect. Disord. 227, 483–493. doi: 10.1016/j.jad.2017.11.048, 29156362

[ref46] Xu Z. Gao F. Fa A. Qu W. Zhang Z. (2024). Statistical power analysis and sample size planning for moderated mediation models. Behav. Res. Methods 56, 6130–6149. doi: 10.3758/s13428-024-02342-2, 38308148

[ref47] Xu D. Jaggars S. S. (2013). The impact of online learning on students’ course outcomes: evidence from a large community and technical college system. Econ. Educ. Rev. 37, 46–57. doi: 10.1016/j.econedurev.2013.08.001

[ref48] Zimmerman B. J. (2000). “Attaining self-regulation: a social cognitive perspective,” in Handbook of Self-Regulation, eds. Boekaerts M. Pintrich P. R. Zeidner M. (San Diego, CA: Academic Press), 13–39.

