# Correction: Associations between test anxiety and academic achievement in distance education: avoidance- and overcontrol-related process and the moderating role of exam format preference

**DOI:** 10.3389/fpsyg.2026.1992612

**Published:** 2026-09-24

**Authors:** Münevver Başman

**Affiliations:** Department of Measurement and Evaluation in Education, Marmara University, Maltepe, Türkiye

**Keywords:** academic perfectionism, academic procrastination, distance education, exam format preference, test anxiety

There was a mistake in Figure 2 as published. In the Author's Proof, the arrow was correctly directed from APER to GPA. However, while preparing a higher-resolution version of Figure 2 as requested during the Author's Proof Corrections, the arrow was incorrectly drawn from AP to APER. The corrected Figure 2 appears below.

**Figure 2:**
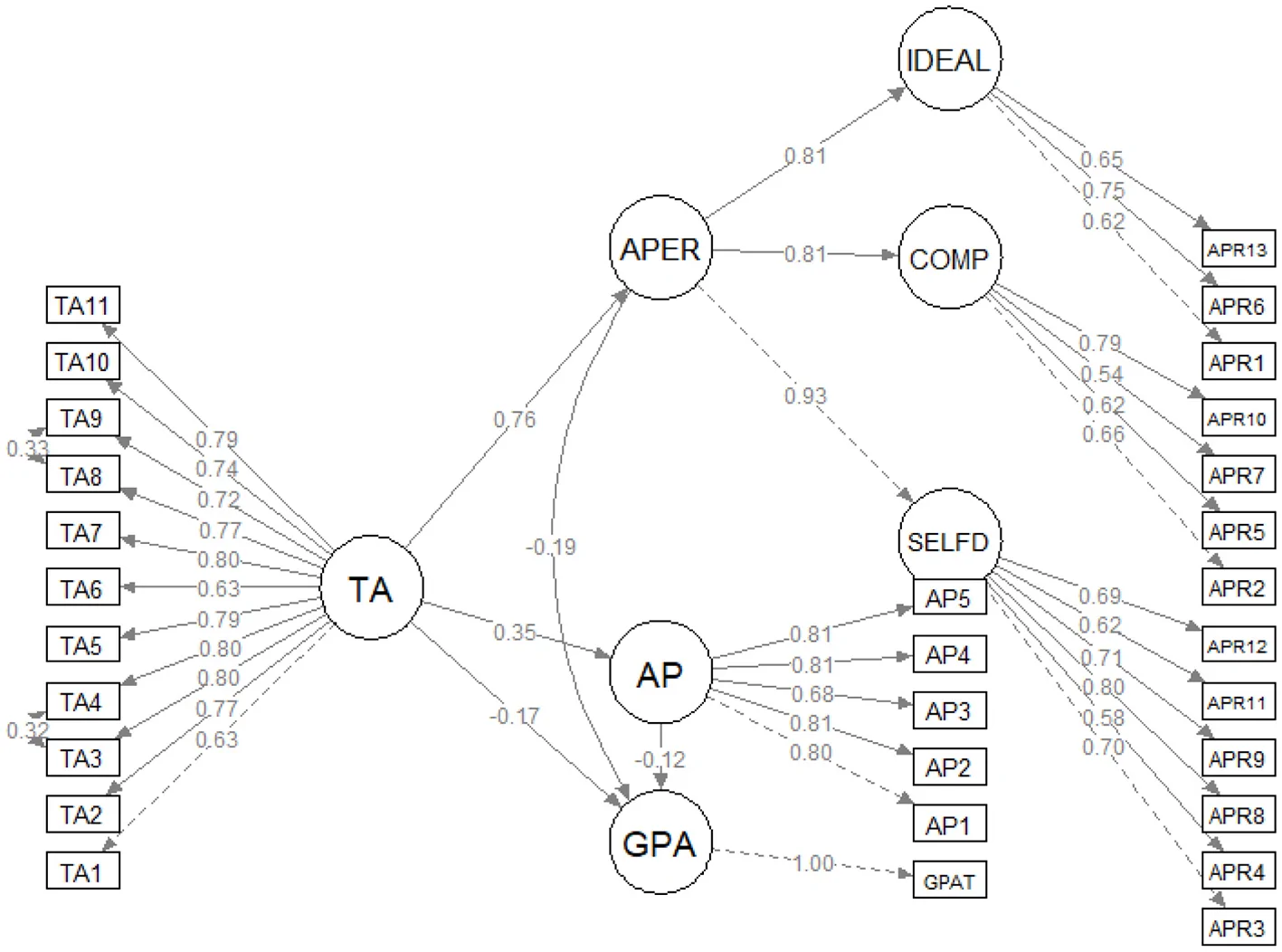
Results of structural model.

The original version of this article has been updated.

